# Impact of Pulmonary Comorbidities on COVID-19: Acute and Long-Term Evaluations

**DOI:** 10.3390/jcm14051446

**Published:** 2025-02-21

**Authors:** Gabriela Mara, Gheorghe Nini, Coralia Cotoraci

**Affiliations:** 1 Multidisciplinary Doctoral School, Vasile Goldis Western University of Arad, 310414 Arad, Romania; mara.gabriela@uvvg.ro; 2 Pneumology Department, Vasile Goldis Western University of Arad, 310414 Arad, Romania; nini.gheorghe@uvvg.ro; 3 Clinical Hematology Department, Vasile Goldis Western University of Arad, 310025 Arad, Romania

**Keywords:** COVID-19, pulmonary comorbidities, chronic obstructive pulmonary disease (COPD), asthma, interstitial lung diseases (ILDs), long COVID, respiratory sequelae, post-acute sequelae of SARS-CoV-2 infection, pulmonary rehabilitation, fibrosis

## Abstract

**Background/Objectives**: Pulmonary comorbidities, such as chronic obstructive pulmonary disease (COPD), asthma, and interstitial lung diseases (ILDs), have emerged as critical factors influencing the severity and outcomes of COVID-19. This review aims to evaluate the interplay between these comorbidities and COVID-19, both during the acute phase and in long-term recovery, focusing on their impact on clinical management and outcomes. **Methods**: This systematic review examined studies sourced from major medical databases, including PubMed and Scopus, using keywords such as “COVID-19”, “pulmonary comorbidities”, “long COVID”, and “respiratory sequelae”. Peer-reviewed articles published from January 2020 to the present were included, with data extracted to evaluate both the acute and long-term effects of these comorbidities on COVID-19 patients. **Results**: Patients with COPD demonstrated significantly higher risks of severe COVID-19, including increased hospitalization and mortality. Asthma, while less consistently associated with severe outcomes, showed a variable risk based on disease control. ILDs were strongly correlated with poor outcomes, including higher rates of respiratory failure and mortality. Long-term complications, such as persistent dyspnea, impaired lung function, and structural changes like fibrosis, were prevalent in patients recovering from moderate to severe COVID-19. These complications adversely affected quality of life and increased healthcare dependency. **Conclusions**: Pulmonary comorbidities amplify both the acute severity and long-term respiratory consequences of COVID-19. Effective management necessitates tailored strategies addressing both phases, integrating rehabilitation and continuous monitoring to mitigate chronic impairments. Future research should prioritize understanding the mechanisms behind these interactions to inform public health interventions and improve patient outcomes.

## 1. Introduction

In December 2019, a new strain of coronavirus, SARS-CoV-2, was first detected in Wuhan, Hubei Province, China. It was identified as the pathogen responsible for a series of unusual respiratory infections, later named COVID-19. Due to its rapid worldwide transmission and profound effects on healthcare systems, the World Health Organization officially classified it as a pandemic on 11 March 2020 [1]. The COVID-19 pandemic has posed major challenges to global health, emphasizing the importance of understanding how pre-existing medical conditions influence disease outcomes [2]. Among these, pulmonary comorbidities, such as chronic obstructive pulmonary disease (COPD), asthma, and interstitial lung diseases, have been recognized as significant contributors to the severity of COVID-19 infections [2]. Additionally, the long-term respiratory complications linked to COVID-19, often referred to as post-COVID-19 conditions or post-acute sequelae of SARS-CoV-2 infection, present further challenges for both healthcare systems and those impacted by the disease [3].

This review aims to provide a thorough analysis of the connection between pulmonary comorbidities and COVID-19 severity, while also examining the long-term respiratory effects of post-COVID-19 conditions. By evaluating existing research, it seeks to clarify how pre-existing respiratory conditions—such as chronic obstructive pulmonary disease (COPD), asthma, and interstitial lung diseases—impact COVID-19 progression and outcomes. Additionally, it highlights the prevalence and nature of persistent respiratory complications, including functional impairments and structural lung changes, that may develop following acute infection. Ultimately, this review aims to synthesize current knowledge to guide public health strategies, improve clinical management for at-risk populations, and identify key areas for future research to address gaps in understanding and care for those affected by COVID-19 and its long-term consequences.

## 2. Methods

The methodology for this review was carefully designed to ensure a comprehensive and structured exploration of the impact of pulmonary comorbidities on COVID-19 severity and the long-term respiratory consequences of post-COVID-19 conditions. A systematic search was conducted across major academic and medical databases, including PubMed, Scopus, and Web of Science. The search strategy utilized specific keywords such as “COVID-19”, “pulmonary comorbidities”, “long COVID”, and “respiratory sequelae”. Articles published between January 2020 and the present were considered to ensure that the analysis reflected the most current knowledge.

## 3. Pulmonary Comorbidities and COVID-19 Severity: Examination of How Chronic Respiratory Diseases Affect Acute COVID-19 Outcomes

Chronic respiratory diseases (CRDs), such as chronic obstructive pulmonary disease (COPD), asthma, and interstitial lung diseases (ILDs), are critical factors influencing the severity and progression of COVID-19 [4]. Patients with pre-existing CRDs often experience more severe clinical outcomes due to the underlying physiological and immune dysfunction associated with these conditions [5]. Understanding these interactions is essential to optimize care and improve prognosis for this vulnerable population.

COPD is one of the most significant pulmonary comorbidities linked to severe COVID-19 [6]. This disease is characterized by chronic inflammation, structural lung damage, and airflow limitation, which reduce the patient’s ability to effectively respond to respiratory infections. Numerous studies have demonstrated that COPD patients with COVID-19 face higher risks of hospitalization, intensive care unit (ICU) admission, mechanical ventilation, and mortality (Table 1).

Asthma, another common CRD, presents a complex relationship with COVID-19 outcomes. While early studies suggested that asthma might not significantly increase the risk of severe disease, more recent data indicate that factors such as airway hyperresponsiveness and chronic inflammation can exacerbate the respiratory distress caused by SARS-CoV-2 [17]. However, well-managed asthma, particularly in patients adhering to maintenance therapy, may not substantially elevate the severity of COVID-19 (Table 2).

ILDs, including idiopathic pulmonary fibrosis (IPF), are also associated with poorer outcomes in COVID-19. ILDs involve chronic inflammation and fibrotic changes in lung tissue, leading to impaired gas exchange and a reduced pulmonary reserve. When infected with SARS-CoV-2, these patients are more likely to experience severe hypoxemia and respiratory failure. Studies have shown that ILD patients with COVID-19 have increased mortality rates compared to those without pre-existing pulmonary fibrosis, highlighting the compounded risks posed by reduced lung capacity and systemic inflammatory responses (Table 3).

## 4. Post-COVID-19 Pulmonary Sequelae: Long-Term Respiratory Complications and Clinical Manifestations

Post-COVID-19 pulmonary sequelae have emerged as a significant concern, particularly for patients who experienced moderate to severe forms of COVID-19. These long-term complications often manifest as persistent respiratory symptoms, functional impairments, and structural abnormalities in the lungs, with substantial implications for quality of life and healthcare systems worldwide (Table 4).

These long-term complications underscore the importance of ongoing research to better understand post-COVID-19 pulmonary sequelae and develop effective therapies. As the pandemic’s impact continues to evolve, addressing these issues will remain a critical component of healthcare systems globally.

## 5. Comparative Analysis: Evaluation of the Relative Impact of Pulmonary Comorbidities During the Acute Phase Versus the Chronic Phase of COVID-19

The interplay between pulmonary comorbidities and COVID-19 manifests differently during the acute and chronic phases of the disease, reflecting distinct challenges in clinical management and long-term care. This comparative analysis aims to evaluate how conditions like chronic obstructive pulmonary disease (COPD), asthma, and interstitial lung diseases (ILDs) influence outcomes during these two phases (Table 5).

The burden of COVID-19 in patients with pulmonary comorbidities varies significantly between the acute and chronic phases of the disease. During the acute phase, the focus is on managing severe inflammatory responses, acute hypoxemia, and the high demand for critical care resources. In contrast, the chronic phase is characterized by lingering respiratory complications, prolonged oxygen dependency, and the need for long-term healthcare interventions. The table below compares these key aspects across the two phases (Table 6).

## 6. Implications for Clinical Management

Managing patients with pulmonary comorbidities who contract COVID-19 requires a tailored approach that addresses the unique challenges of both the acute and chronic phases of the disease. During the acute phase, the primary focus is on mitigating severe complications, such as respiratory failure and thrombotic events, while ensuring optimal control of underlying lung conditions. In the post-acute and chronic phases, attention shifts to long-term recovery, rehabilitation, and the management of persistent symptoms and pulmonary sequelae. The table below outlines key strategies for each phase of clinical management (Table 7).

Oxygen therapy is essential for managing respiratory complications in COVID-19, especially in patients with COPD and idiopathic pulmonary fibrosis. Severe cases often require high-flow nasal oxygen, non-invasive ventilation, or mechanical ventilation [26]. Post COVID-19, long-term oxygen therapy may be needed due to persistent fibrosis and diffusion impairment, improving functional outcomes and reducing hospital readmissions [46]. However, the optimal duration and monitoring continue to be debated.

There have been advancements in AI support clinical management, with a wrapper-based deep learning technique achieving 97.7% accuracy in detecting chest infections, including COVID-19, from X-rays. By combining deep feature extraction, ten optimization algorithms, and a support vector machine, this method enhances diagnostic precision and aids early detection and decision-making for pulmonary comorbidities [47].

## 7. Conclusions

This review highlights the critical influence of pulmonary comorbidities, such as COPD, asthma, and interstitial lung diseases, on the severity and outcomes of COVID-19. Patients with these conditions are more susceptible to severe complications during the acute phase, including increased hospitalization and mortality rates. Furthermore, the long-term effects, characterized by persistent respiratory symptoms, functional impairments, and structural lung changes, add substantial burden to survivors. The review underscores the necessity for tailored clinical management and ongoing research to better understand and address the complex interplay between COVID-19 and pre-existing pulmonary diseases, aiming to inform future healthcare strategies and improve patient outcomes.

## Figures and Tables

**Table 1:** COPD and COVID-19 clinical outcomes analysis.

Country	Study Type	Total Participants	Main Reported Outcomes/Analyses	References
SUA	Longitudinal Cohort Study	157 patients	Assessed the physical and mental health of COPD patients during the COVID-19 pandemic; found low incidence of COVID-19 infection and stable levels of anxiety and depression among participants. Elevated anxiety (8%) and depressive symptoms (19%) in June 2020 remained stable throughout follow-up.	[7]
SUA	Retrospective Cohort Study	31,526 patients	Evaluated outcomes of COPD patients hospitalized with COVID-19; Mortality was higher in COPD patients (14.02%) compared to those without COPD (8.8%); COPD mortality risk varied by sex and age, being higher in females (OR 1.62, 95% CI 1.36–1.95) but not in males. Increased risk was also noted in patients aged 40–64 (OR 1.42, 95% CI 1.07–1.90) and 65–79 (OR 1.48, 95% CI 1.23–1.78) years.	[8]
SUA	Retrospective Study	577 patients, of which 103 were diagnosed with COPD/emphysema	Investigated the impact of COPD and emphysema on hospitalized COVID-19 patients; concluded that pre-existing COPD and radiographic evidence of emphysema had higher rates of intensive care unit (ICU) admission (35% versus 24.9%) and maximal respiratory support requirements, with more frequent invasive mechanical ventilation (21.4% versus 11.8%), but no significant difference in mortality.	[9]
Global	Systematic Review and Meta-Analysis	123 studies	COPD prevalence in patients hospitalized with COVID-19 varies markedly by region (3.82 to 11.85%); COPD was associated with increased mortality risk 1.863 (95% CI 1.640–2.115).	[10]
Spain	Retrospective Case–Control Study	120 patients	Evaluated the impact of COVID-19 on COPD exacerbations and clinical course; found that COVID-19 led to increased exacerbations and worse clinical outcomes in COPD patients compared to non-COVID-19 periods.	[11]
China	Multicenter, Retrospective, Observational Study	1048 patients of which 50 were diagnosed with COPD	Patients with COPD showed a higher risk of developing bacterial/fungal coinfection (20.0% vs. 5.9%), acute respiratory distress syndrome (ARDS) (20.0% vs. 7.3%), septic shock (14.0% vs. 2.3%), or acute renal failure (12.0% vs. 1.3%); and there was a risk of death (HR: 2.28, 95% CI: 1.15–4.51; *p* = 0.019).	[12]
Denmark	Cohort Study	5104 patients, of which 432 were diagnosed with COPD	The standardized absolute risk of the combined end-point was 21.2% (95% CI 18.8–23.6). Low blood eosinophil counts (<0.3 × 10^9^ cells·L^−1^) were associated with increased risk of severe outcomes among patients with COPD.	[13]
Global	Systematic Review and Meta-Analysis	100 studies (n = 1,229,434 patients)	COPD significantly increased the mortality risk (OR 3.79, *p* < 0.001).	[14]
Latin America	Systematic Review and Meta-Analysis	Multiple studies	COPD prevalence in hospitalized COVID-19 patients varies significantly across Latin American countries; post-COVID-19 pulmonary fibrosis remains a major challenge due to limited healthcare resources and access to specialized care.	[15]
Africa	Observational Study	Multiple studies and reports	COVID-19 worsened lung health outcomes in Africa, particularly for patients with pre-existing chronic respiratory diseases (CRDs) such as COPD. Limited access to healthcare, a lack of diagnostic tools (e.g., spirometry), and a redirection of resources to COVID-19 response led to underdiagnosis and undertreatment of these conditions. Additionally, exposure to environmental pollutants, tobacco smoke, and high rates of tuberculosis further increased the severity of COVID-19 in African populations.	[16]

**Table 2:** Asthma and COVID-19 clinical outcomes analysis.

Country	Study Type	Total Participants	Main Reported Outcomes/Analyses	References
Hong Kong	Retrospective Cohort Study	74,396, of which 1290 were diagnosed asthma patients	The rates of death and the composite outcome were 15.3% and 17.2%, respectively, and among the non-asthma patients, they were 12.2% and 13.6%, respectively. Asthma reduced disease severity risk despite incomplete vaccination.	[18]
Japan	Retrospective Multivariate Analysis	72,582 patients, of which 3731 were diagnosed with asthma	Asthma showed no significant association with an increase in mortality (OR 0.837, 95% CI 0.639–1.080, *p* = 0.184) or invasive mechanical ventilation events (OR 1.084, 95% CI 0.878–1.326, *p* = 0.440).	[19]
UK	Retrospective Study	35,202,533 adults and 2,996,503 children	Adults with asthma on medium or high-dose inhaled corticosteroids had a higher risk of COVID-19 death (HRs 1.18 and 1.36). Hospitalization risk was higher for children with asthma who were prescribed one (2.58) or multiple (3.80) corticosteroid courses pre-pandemic.	[20]
UK	Mixed Methods Analysis	4500 people diagnosed with asthma	Among asthma patients with COVID-19 (10.5%, n = 471), 56.1% reported long COVID. This group experienced worsened breathing (73.7% vs. 34.8%), increased inhaler use (67.8% vs. 34.8%), and poorer asthma control (59.6% vs. 25.6%) (*p* < 0.001).	[21]
Global	Systematic Review and Meta-Analysis	90 studies	Asthma patients with COVID-19 had a slightly higher hospitalization risk (RR 1.13) but no increased risk for severe disease, ICU admission, ventilation, or death.	[17]
Global	Systematic Review and Meta-Analysis	30 (n = 112,420) studies	Asthma was linked to lower risk for all outcomes, but evidence was very uncertain (95% CIs included no difference or increased risk). Hospitalization data showed high heterogeneity. Allergic asthma lowered COVID-19 risk, while COPD increased it.	[22]
Global	Systematic Review and Meta-Analysis	100 studies (n = 1,229,434 patients)	Asthma had no significant impact on COVID-19 mortality (OR 0.89, *p* = 0.630).	[14]
Global	Systematic Review and Meta-Analysis	123 studies	Asthma prevalence was 4.21 to 12.39%; the relative risk of death for asthma was 0.918 (95% CI 0.767 to 1.098) with no evidence of increased mortality.	[10]
Denmark	Cohort Study	5104 patients, of which 354 were diagnosed with asthma	The standardized absolute risk of the combined end-point was 18.5% (95% CI 14.3–22.7) in patients with asthma.	[13]

**Table 3:** ILDs and COVID-19 clinical outcomes analysis.

Country	Study Type	Total Participants	Main Reported Outcomes/Analyses	References
USA	Multicenter study	311,060 patients, of which 251 patients (0.08%) were diagnosed with IPF	IPF patients (mean age 68.3, 56.97% male) had more comorbidities than non-IPF patients. After matching, they had higher 30- and 60-day mortality or ventilation rates, hospitalization, critical care needs, and acute kidney injury.	[23]
UK	Multicenter study	349 ILD patients	Mortality in ILD patients with COVID-19 was 49% (79/161). Death risk was higher with FVC < 80% (HR 1.72) and obesity (HR 2.27).	[24]
Global	Systematic review and meta-analysis	15 studies with 135,263 patients, of which 1.4% were diagnosed with ILD	ILD prevalence in COVID-19 patients was 1.4%. ILD was more common in non-survivors (RR 2.728) and increased mortality (RR 2.454) and ICU admission risk (RR 3.064). Mechanical ventilation rates showed no difference.	[25]
Spain	Multicenter study	137 patients diagnosed with ILD	IPF patients tended to have ILD worsening (60%, *p* = 0.12), while other ILD patients showed a trend toward recovery (63.4%, *p* = 0.124)	[26]
Japan	Multicenter study	937,758, of which 7333 (0.8%) had pre-existing ILD	ILD prevalence declined across waves (wild-type 1.2%, alpha 0.8%, delta 0.3%), as did 60-day mortality (16.0%, 14.6%, 7.5%). Mortality significantly decreased from alpha to delta (−7.1%, 95% CI −9.3% to −4.9%). ILD was independently linked to higher mortality in all waves (OR ~2.10).	[27]
Japan	Clinical study	26 ILD patients	Severe cases (23.1% vs. 42.3%, *p* = 0.114) and mortality (11.5% vs. 3.8%, *p* = 0.326) were higher in the ILD group, though not statistically significant.	[28]
Japan	Multicentric study	854 patients with acute exacerbation of ILD	Hospitalizations increased at 17 hospitals, decreased at 27, and remained unchanged at 88. COVID-19-related exacerbations had worse 30- and 90-day mortality than non-COVID-19 ones.	[29]
Australia	Multicentric study	145 COVID-19 patients, with 133 reassessed after 100 days	41% had persistent symptoms at 100 days; 63% showed radiological lung abnormalities (GGOs, consolidation, reticulation). Most patients experienced symptom and lung improvement within 3 months.	[30]
Italy	Retrospective study	90 patients	Pulmonary fibrosis developed in 23 patients (25.5%), mostly older males (75 ± 15 years), with high rates of smoking (60.8%) and comorbidities, particularly hypertension (47.8%) and diabetes (34.7%).	[31]
Norway	Prospective cohort study	103 participants, including 15 ICU patients	One in four patients had persistent lung abnormalities and impaired gas exchange 3 months following hospital admission for COVID-19. ICU admission was linked to more CT abnormalities but did not worsen lung function or dyspnoea.	[32]

**Table 4:** Key aspects of post-COVID-19 pulmonary sequelae.

Category	Key Findings	Examples/Details	References
Persistent Symptoms	Long-term respiratory issues reported by patients.	- Dyspnea: Common among survivors; linked to impaired gas exchange and reduced lung capacity. - Chronic Cough: Often due to residual airway inflammation. - Fatigue: Multifactorial, often coexisting with respiratory impairments.	[33]
Pulmonary Function	Impairments detected in lung function tests.	- Reduced DLCO: Indicates impaired alveolar gas exchange. - Restrictive Deficits: Reduced lung volumes linked to fibrosis. - Obstructive Patterns: Rare but observed in pre-existing airway diseases.	[34,35]
Radiological Changes	Persistent abnormalities on imaging studies.	- Ground-glass opacities (GGOs): Common and linked to lingering inflammation. - Pulmonary Fibrosis: Scarring, especially in severe cases. - Bronchiectasis: Irreversible airway dilation.	[36,37,38]
Pathophysiology	Underlying mechanisms driving sequelae.	- Persistent Inflammation: Chronic low-grade inflammation. - Endothelial Dysfunction: Contributing to impaired gas exchange. - Immune Dysregulation: Ongoing tissue damage.	[39,40]
Management Strategies	Approaches to mitigate and treat post-COVID-19 pulmonary complications.	- Pulmonary Rehabilitation: Exercises and breathing techniques to restore function. - Pharmacotherapy: Antifibrotic drugs (e.g., nintedanib, pirfenidone). - Monitoring: Regular imaging and PFTs.	[41]
Clinical Outcomes	Overall impact on recovery and quality of life.	On recovery and quality of life: - Reduced physical and functional capacity. - Prolonged recovery time. - Increased healthcare utilization for follow-ups and treatments.	[42]

**Table 5:** Comparative impact of pulmonary comorbidities on acute and chronic phases of COVID-19.

Pulmonary Comorbidity	Acute Phase Impact	Chronic Phase Impact
COPD	- Severity Increase: 1.9× higher mortality risk in hospitalized patients [43]. - Exacerbations: Triggered by inflammation and viral load, leading to worsened airflow limitation.	- Chronic Symptoms: Dyspnea, fatigue, and reduced exercise tolerance persist for months post-recovery. - Functional Decline: Reduced DLCO and long-term pulmonary impairment [44].
Asthma	- Variable Severity: Generally less severe impact compared to COPD, but severe asthma increases hospitalization risk.	- Persistent Symptoms: Prolonged respiratory distress in severe cases; overall lower rates of fibrosis than COPD or ILDs.
ILDs	- Exacerbations: Acute COVID-19 often triggers exacerbations leading to respiratory failure [36]. - Severe Outcomes: High ICU admission rates due to compromised lung function.	- Fibrosis Progression: Accelerated fibrosis in pre-existing ILD patients, worsening prognosis [37]. - Chronic Symptoms: Persistent dyspnea, fatigue, and reduced lung capacity.
General Observations	- Increased Mortality: Across all comorbidities, higher ICU and mechanical ventilation rates. - Immune Defenses: Baseline impairment leads to greater susceptibility to severe disease.	- Post-COVID-19 Sequelae: Persistent respiratory symptoms, fibrosis development, and reduced functional capacity, varying by baseline pathology severity [45].

**Table 6:** Disease burden in acute and chronic phases of COVID-19 in patients with pulmonary comorbidities.

Aspect	Acute Phase	Chronic Phase
Inflammatory Burden	- Cytokine storm disproportionately impacts pre-existing pulmonary conditions. - Rapid clinical deterioration due to intense inflammation.	- Lingering inflammation and immune dysregulation. - Prolonged symptoms and structural lung damage.
Oxygen Dependency	- Acute hypoxemia caused by viral pneumonia and ARDS. - High oxygen demand requiring ICU care.	- Persistent oxygen dependency in cases with pre-existing fibrosis or significant lung scarring.
Healthcare Utilization	- Increased hospitalization and ICU admission. - Need for mechanical ventilation in severe cases.	- Frequent follow-ups and long-term pulmonary rehabilitation. - Advanced therapies to manage sequelae.

**Table 7:** Key strategies for managing each phase of clinical care for COVID-19 patients with pulmonary comorbidities.

Phase	Key Strategies	Details
Acute Phase Management	Early Identification and Risk Stratification	Identify high-risk patients (e.g., those with COPD, asthma, ILDs, advanced age, or smoking history). Monitor oxygen saturation and respiratory function closely.
	Optimizing Pre-existing Condition Management	Ensure adequate control of underlying pulmonary diseases by continuing prescribed therapies like inhaled corticosteroids and bronchodilators to reduce the risk of exacerbations.
	Use of Antiviral and Immunomodulatory Therapies	Administer antivirals (e.g., remdesivir, molnupiravir) and immunomodulatory drugs (e.g., dexamethasone, tocilizumab) to reduce viral replication and inflammation.
	Oxygen Therapy and Mechanical Ventilation	Provide supplemental oxygen or advanced respiratory support (e.g., HFNC, NIV). In severe cases, invasive mechanical ventilation or ECMO may be necessary.
	Anticoagulation Management	Use thromboprophylaxis to mitigate the high risk of thrombosis in COVID-19 patients, especially those with limited mobility and pulmonary comorbidities.
Post-Acute and Chronic Phase Management	Comprehensive Post-COVID-19 Evaluation	Conduct regular follow-ups with pulmonary function tests (PFTs), chest imaging (e.g., CT scans), and assessments of exercise tolerance to detect and monitor post-COVID-19 sequelae like fibrosis and bronchiectasis.
	Management of Long-Term Symptoms	Address persistent symptoms (e.g., dyspnea, fatigue, chronic cough) with targeted interventions. Use short-term corticosteroids for inflammation and antifibrotic therapies (e.g., nintedanib, pirfenidone) for fibrosis.
	Pulmonary Rehabilitation	Implement rehabilitation programs with breathing exercises, graded physical activity, and psychological support to improve lung function and manage post-traumatic stress and anxiety.
	Nutritional and Psychological Support	Optimize nutrition to aid recovery in debilitated patients. Provide mental health support for anxiety and depression, especially for those with prolonged illness.
